# Supplementary material for: Geometric, Electronic, and Optoelectronic Properties of Carbon-Based Polynuclear C3O[C(CN)2]2M3 (where M = Li, Na, and K) Clusters: A DFT Study
Source: Molecules. 2023 Feb 15;28(4):1827. doi: 10.3390/molecules28041827 (PMC9965956; doi:10.3390/molecules28041827)
Supplement: Supplementary file 1 [file molecules-28-01827-s001.zip › molecules-2166131-supplementary.pdf]

Supplementary information

Geometric, electronic, and optoelectronic properties of carbon-based polynuclear $C_3O[C(CN)_2]_2M_3$ (where M=Li, Na, and K) clusters; A DFT study

Imene Bayach[1*†], Atazaz Ahsin[2†], Safi Ullah Majid[2], Umer Rashid[2], Nadeem S. Sheikh[3], and Khurshid Ayub[2*]

[1] Department of Chemistry, College of Science, King Faisal University, Al-Ahsa, 31982, Saudi Arabia
[2] Department of Chemistry, COMSATS University Islamabad, Abbottabad Campus, Abbottabad, KPK, 22060, Pakistan
[3] Chemical Sciences, Faculty of Science, Universiti Brunei Darussalam, Jalan Tungku Link, Gadong BE1410, Brunei Darussalam

*Correspondence: ibayach@kfu.edu.sa (I.B.), khurshid@cuiatd.edu.pk (K.A)
[†] These authors contributed equally.

Table S1: Optimized geometric coordinates C1 to C3 superalkali clusters at CAM/B3LYP

C1

1	6	0	0.000000	0.700942	0.364055
2	6	0	0.000000	0.000000	1.546169
3	6	0	0.000000	-0.700942	0.364055
4	6	0	0.000000	1.895285	-0.371518
5	6	0	0.000000	-1.895285	-0.371518
6	6	0	0.000000	1.799737	-1.761650
7	6	0	0.000000	3.128695	0.320051
8	6	0	0.000000	-3.128695	0.320051
9	6	0	0.000000	-1.799737	-1.761650
10	7	0	0.000000	4.090874	0.960869
11	7	0	0.000000	1.598177	-2.905338
12	7	0	0.000000	-1.598177	-2.905338
13	7	0	0.000000	-4.090874	0.960869
14	3	0	0.000000	1.317819	4.125301
15	3	0	0.000000	-1.317819	4.125301
16	3	0	0.000000	0.000000	-3.998176
17	8	0	0.000000	0.000000	2.822124

C2

1	6	0	0.000000	0.693494	0.014382
2	6	0	0.000000	0.000000	-1.180430
3	6	0	0.000000	-0.693494	0.014382
4	6	0	0.000000	1.962486	0.627117
5	6	0	0.000000	-1.962486	0.627117
6	6	0	0.000000	2.042327	2.016813
7	6	0	0.000000	3.019979	-0.305522
8	6	0	0.000000	-3.019979	-0.305522
9	6	0	0.000000	-2.042327	2.016813
10	7	0	0.000000	3.710567	-1.236990
11	7	0	0.000000	1.942208	3.175058
12	7	0	0.000000	-1.942208	3.175058
13	7	0	0.000000	-3.710567	-1.236990
14	8	0	0.000000	0.000000	-2.435411
15	11	0	0.000000	-1.978327	-3.519353
16	11	0	0.000000	1.978327	-3.519353
17	11	0	0.000000	0.000000	4.420473

C3

1	6	0	0.000000	0.693470	0.109005
2	6	0	0.000000	0.000000	-1.091788
3	6	0	0.000000	-0.693470	0.109005
4	6	0	0.000000	1.954149	0.744203
5	6	0	0.000000	-1.954149	0.744203
6	6	0	0.000000	2.045815	2.135388
7	6	0	0.000000	3.051061	-0.139660
8	6	0	0.000000	-3.051061	-0.139660
9	6	0	0.000000	-2.045815	2.135388

10	7	0	0.000000	3.825056	-1.003607
11	7	0	0.000000	2.012238	3.297138
12	7	0	0.000000	-2.012238	3.297138
13	7	0	0.000000	-3.825056	-1.003607
14	8	0	0.000000	0.000000	-2.342879
15	19	0	0.000000	-2.267081	-3.578856
16	19	0	0.000000	2.267081	-3.578856
17	19	0	0.000000	0.000000	4.999666

Density of states (DOS) analysis

For the further confirmation of electronic properties, we carried out total density of states study and their spectra are given in Figure S1. The plotted TDOS plots show the change in number of states per unit energy; these spectra are plotted between the energy ranges of –20 to 20 eV. One can observe the newly generated number of states at higher energy and reduced HOMO-LUMO gaps for **C1** to **C3** clusters. The shifting of HOMO peaks to higher energies suggest the improved electrical and conductive properties of these clusters. The shifted HOMO peak to higher energy is due to bigger size of alkali metals which is dominant factor in controlling conductive and electronic properties of designed clusters. In cluster **C3**, the narrowing E_{H-L} can be seen due to generation of new energy HOMO levels at higher energy.

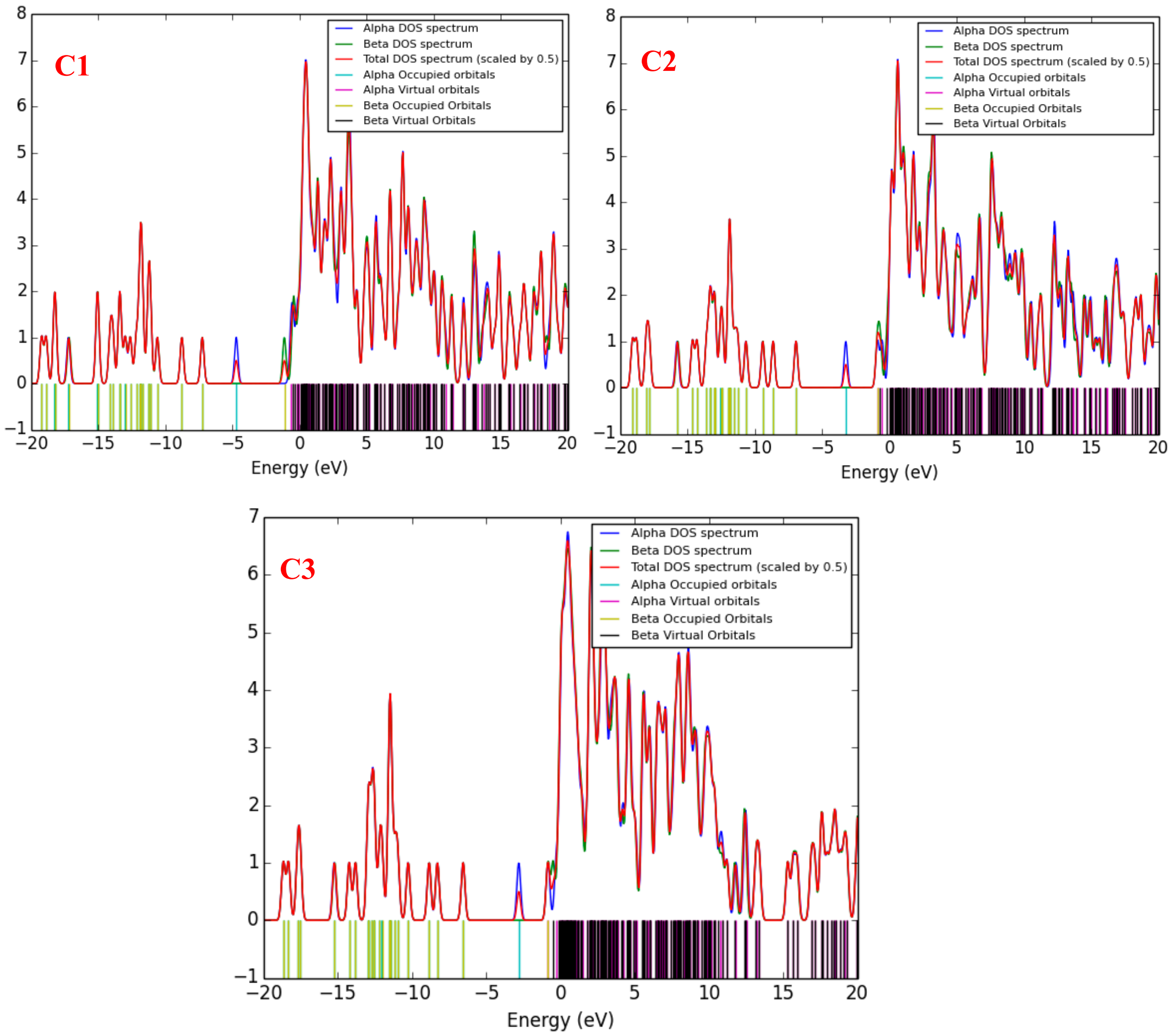

Figure S1: The DOS spectra of studied compound
